# Factors associated with nutritional status of infants and young children in Somali Region, Ethiopia: a cross- sectional study

**DOI:** 10.1186/s12889-015-2190-7

**Published:** 2015-09-02

**Authors:** Yirgu Fekadu, Addisalem Mesfin, Demewoz Haile, Barbara J. Stoecker

**Affiliations:** Applied Human Nutrition Department, School of Nutrition, Food Science and Technology, Hawassa University, Hawassa, Ethiopia; Department of Reproductive Health, College of Medicine and Health Sciences, Bahir Dar University, P.O. Box 79, Bahir Dar, Ethiopia; Department of Nutritional Sciences, Oklahoma State University, Stillwater, OK 74074 USA

## Abstract

**Background:**

Inadequate nutrition during the first two years of life may lead to childhood morbidity and mortality, as well as inadequate brain development. Infants are at increased risk of malnutrition by six months, when breast milk alone is no longer sufficient to meet their nutritional requirements. However the factors associated with nutritional status of infants after 6 months of age have received little attention in pastoralist communities of Ethiopia. Therefore this study aimed to identify the factors associated with nutritional status of infants and young children (6–23 months) in Filtu town, Somali Region, Ethiopia.

**Methods:**

A cross-sectional community-based study was conducted. Simple random sampling was employed to select 214 infants for the study. Univariable and multivariable logistic regressions models were used in the statistical analysis. The strength of association was measured by odds ratios with 95 % confidence intervals. Both the crude (COR) and adjusted odds ratios (AOR) are reported.

**Results:**

The prevalence of wasting, stunting and underweight among infants and young children were 17.5 % (95 % CI: 12.91-23.22), 22.9 % (95 % CI: 17.6-28.9) and 19.5 % (95 % CI: 14.58-25.3) respectively. The multivariable logistic regression model showed that breastfeeding was independently associated with reduced odds of wasting (AOR = 0.38(95 % CI: 0.14-0.99)). Diarrhea in the past 15 days (AOR = 2.13 (95 % CI: 1.55-4.69)) was also associated with increased odds for wasting. The independent predictors of reduced odds for stunting were dietary diversity score ≥4 (AOR = 0.45(95 % CI: 0.21-0.95)) and introduction of complementary feeding at 6 months (AOR = 0.25 (95 % CI: 0.09-0.66)). Bottle feeding was associated with increased odds of stunting (AOR = 3.83 (95 % CI: 1.69-8.67)). Breastfeeding was associated with reduced odds of underweight (AOR = 0.24 (95 % CI: 0.1-0.59)), while diarrheal disease in the past 15 days was associated with increased odds of underweight (AOR = 3.54 (95 % CI: 1.17-7.72)).

**Conclusion:**

Under nutrition is a public health problem among infants and young children in Filtu town, Somali region Ethiopia. Breastfeeding was associated with lower odds of wasting and underweight while diarrheal disease was associated with higher odds of wasting and underweight. Low dietary diversity scores, inappropriate age of complementary feeding initiation and bottle feeding were identified to be significant predictors of stunting. Those factors should be considered for any intervention aimed to reduce under nutrition among infants and young children in Filitu town, Somali region, Ethiopia.

## Background

World Health Organization (WHO) and United Nations Children’s Fund (UNICEF) have advocated for increased commitment to appropriate feeding practices for all infants and young children in order to achieve optimal growth, development and health [1]. As global public health recommendations, international guidelines stress that infants should be exclusively breastfed for six months, then frequent and on demand breastfeeding should continue to 24 months and should be coupled with the gradual introduction of complementary feeding adapted to the child’s requirements [2]. This recommendation also applies to Africa. Infants and young children need special attention in order to attain their nutritional requirements as the period of complementary feeding is particularly vulnerable to nutritional deficiencies due to rapid growth [3].

According to a UNICEF report, five infectious diseases (pneumonia, diarrhea, malaria, measles, and AIDS) account for more than one-half of all deaths in children aged <5 years, most of whom are undernourished [4]. Malnutrition increases diarrhea incidence and duration [5, 6]. Moreover malnutrition increases the risk of mortality from diarrheal and acute lower respiratory infection in children < 2 years of age [7]. About 53 % of under five mortality is attributed to malnutrition [8]. Inadequate nutrition during the first two years of life may lead to childhood morbidity and mortality, as well as inadequate brain development [9]. Infants are at increased risk of malnutrition starting from six months, when breast milk alone is no longer sufficient to meet all the nutritional requirements of infants. Children living in most developing countries are introduced directly to the regular household diet made of cereal or starchy root crops which is a major cause for the high incidence of child malnutrition, morbidity and mortality [10–12].

According to the 2011 Ethiopian Demographic and Health Survey (EDHS), in the Somali region the prevalence of stunting, wasting and underweight were 33, 22.2 and 33.5 % respectively for under five year old children [13]. Wasting prevalence in the region is the highest among the regions of the country [13]. Infant and young child feeding practices in the region were reported to be poor as compared to the other regions of Ethiopia. Median duration of breast feeding (16.7 months) was the lowest in the country while only 0.8 and 11.7 % of the children met the guidelines for dietary diversity and minimum meal frequency respectively [13]. However there is a paucity of evidence regarding the factors associated with nutritional status of infants and young children in pastoralist areas in Ethiopia. Therefore the objective of this study was to identify factors associated with nutritional status among infants and young children in Filtu town, Somali region of Ethiopia.

## Methods

### Study setting and design

This community-based cross-sectional study was conducted in Filtu town, which is the capital town of Liben Zone of the Somali Region. The total population in Filtu woreda was 130, 912, of which 4960 reside in filtu town [14]. The climate in Filtu is arid and the rainfall pattern is bimodal with low annual rainfall [15]. Most residents are dependent on animal products (their own or from the market) for consumption. Likewise, most of their income depends on animals, either selling their products or depending on them as a means of income generation like fetching water. A few members of the community were merchants, selling materials from the border of the country. As a whole, the communities have ration distribution from the government through the “work for food” program within their kebeles (smallest administrative unit).

### Sample size and sampling procedure

The sample size was calculated based on a single population proportion formula. Prevalence of malnutrition in the specific age group (6–23 months) of Somali region was used to calculate the sample size. The prevalence of underweight, stunting and wasting were 24.2, 22.3 and 17.8 %, respectively, reported by the 2011 EDHS for the Somali region [13]. The confidence level 95 % (*a* = 0.05) and margin of error (*d* = 0.05) were considered. The largest sample size was taken after sample size was calculated based on the three indicators of under nutrition. A sample size of 276, calculated based on the prevalence of underweight, was found to be the largest and was taken as a sample size for this study. Since the source population was less than 10,000, a finite population correction formula was used and 5 % non-response rate was added to get the final sample size. The total numbers of eligible study subjects in the town were 781 infants and young children.

So considering, *N* = 781, *no* = 276

Finite population correction formula $n=\frac{no}{1+\frac{no}{N}}$

where n_0_ is the total number of the target population (source population). Therefore, after adjusting the initial calculated sample size and adding 5 % of non- response rate, the final sample size was 214.

There are three kebeles in the study area and infants and young children from all the three kebeles were included in the study. All households with infants and young children aged between 6–23 months were registered, and the number for the sample population was allocated according to size of the kebeles. Simple random sampling was employed to select households within each kebele. In those households having more than one infant or young child between 6 and23 months, the index child was selected by the lottery method.

### Data collection procedures

A structured questionnaire adapted from the Ethiopian Demographic and Health Survey (EDHS) was used to collect socio-demographic and other relevant child and mother related information. Besides to the EDHS questionnaire, we included additional questions on the questionnaire for this study based on the study objective. Feeding practices were assessed using a qualitative 24-h dietary recall method. The three data collectors were certified at the diploma level in nursing and spoke the local language fluently. The data collectors were trained on data collection techniques for two days including practical work. Data collectors interviewed each mother individually using the *Somalifa* language version of the questionnaire.

### Measurements

Anthropometric measurements (weight and length) were taken for all children by the principal investigator with an assistant. Standard anthropometric measurement procedures were used as outlined in the measurement guide prepared by the Food and Nutrition Technical Assistance (FANTA) project [16].

The family’s food security status was measured with the household hunger scale and categorized as food secure or food insecure. The household hunger scale is most appropriate to use in areas of substantial food insecurity. Households that scored as “no hunger” to little hunger in the household (score of 0–1) were classified as food secure and moderate and severe hunger in the household were categorized as food insecure (score of 2–6) [17]. Meal frequency and dietary diversity were assessed by 24 h recall. Minimum meal frequency was fulfilled if food was received 2 to 3 times per day at 6 to 8 months of age, 3–4 times per day at age 9–11 months and 3–4 times at age 12 to 24 months, with additional nutritious snacks offered 1–2 times per day between meals in the last 24 h. Minimum dietary diversity was fulfilled if a child had received foods from 4 or more food groups from the seven WHO food groups in the last 24 hrs [18].

Bottle feeding practices were measured by a 24-hour recall as recommended by WHO [18] and the questions was asked as “Did [Child Name] drink anything from a bottle with a nipple yesterday during the day or night time?” Similarly breastfeeding practice was assessed by asking the mother to recall whether her child had breastfed in the last 24 h or not and it was asked as “Did [Child Name] breastfeed yesterday during the day or night time? Wealth index was constructed using household asset data via a principal components analysis to categorize individuals into wealth tertiles (low, medium, high).

### Pre test

The questionnaire was pre-tested to assess for clarity, understandability and completeness in communities which have similar geographic setting and socio demographic profile as the study area. During the pre-test, problems on the order, response options and difficult sentence constructions were identified. Based on the findings of the pre-test, rearrangement of sequence and change of wording of questions was made as needed.

### Data analysis

Anthropometric data were standardized for age using WHO Anthro v. 3.2.2. The data analysis was performed using SPSS version 20. Descriptive statistics (mean and standard deviation) were calculated for continuous variables. The nutritional status indicators, weight-for-length (WLZ), length-for-age (LAZ) and weight-for-age (WAZ) were compared with reference data from World Health Organization standards [3]. Children below-2 standard deviations (−2SD) of the WHO median for WLZ, LAZ, and WAZ were considered wasted, stunted or underweight respectively.

Univariable analysis (bivariate logistic regression) was carried out between the predictor and outcome variables. Using significant variables at p value 0.05 from the binary logistic regression models, a multivariable logistic regression model was fitted to identify the independent predictors of nutritional status (measured as wasting, underweight and stunting). The strength of association was measured by odds ratios with 95 % confidence intervals. Both the Crude (COR) and Adjusted Odds Ratios (AOR) are reported. Variables with *p* < 0.05 in the multivariable logistic regression model were considered as associated factors.

### Ethical consideration

Ethical clearance was obtained from Hawassa University Institutional Review Board. After explaining the study procedures for the study subjects, verbal consents were obtained from each mother/caregiver.

## Results

### Socio-demographic characteristics

Four of the sampled infant-mother pairs were unwilling to participate in the study making a response rate of 98 %. The mean (±SD) age of infants and young children who participated was 15.4 (±6.0) months. From the total of 210 infants and young children, 51.9 % were males and 48.1 % were females.

Almost all of the mothers in the study were married (98.1 %) and the husbands were the heads of the household. A large proportion (42.4 %) of household heads had no education and 15.2 % were unemployed. Among the mothers, 71.4 % had no formal education and 78.6 % were not engaged in work outside of the home (Table 1).

**Table 1:** Socio-demographic characteristics of mothers and household heads (*n* = 210) in Filtu town, Somali Region, April 2013

Socio-demographic characteristics	Frequency	Percentage (%)
Age of the mother(years)		
< 24	68	32.4
25–29	63	30.0
≥ 30	79	37.6
Religion		
Muslim	207	98.6
Orthodox	2	1.0
Protestant	1	0.5
Ethnic group		
Somali	204	97.1
Oromo	3	1.4
Other	3	1.4
Marital status		
Married	206	98.1
Widowed	1	0.5
Separated	3	1.4
Educational status of mothers		
None	150	71.4
Read/write	3	1.4
1–4	17	8.1
5–8	25	11.9
9–12	15	7.1
Occupation of mothers		
Government employees	10	4.8
Petty trading	30	14.3
Unemployed	165	78.6
Others	5	2.4
Educational status of HH		
None	89	42.4
Read/write	5	2.4
1–4	15	7.1
5–8	48	22.9
9–12	53	25.2
Family size Mean (± SD) 7.36(3.11)		

### Nutritional status of the infants and young children

**Fig. 1:**
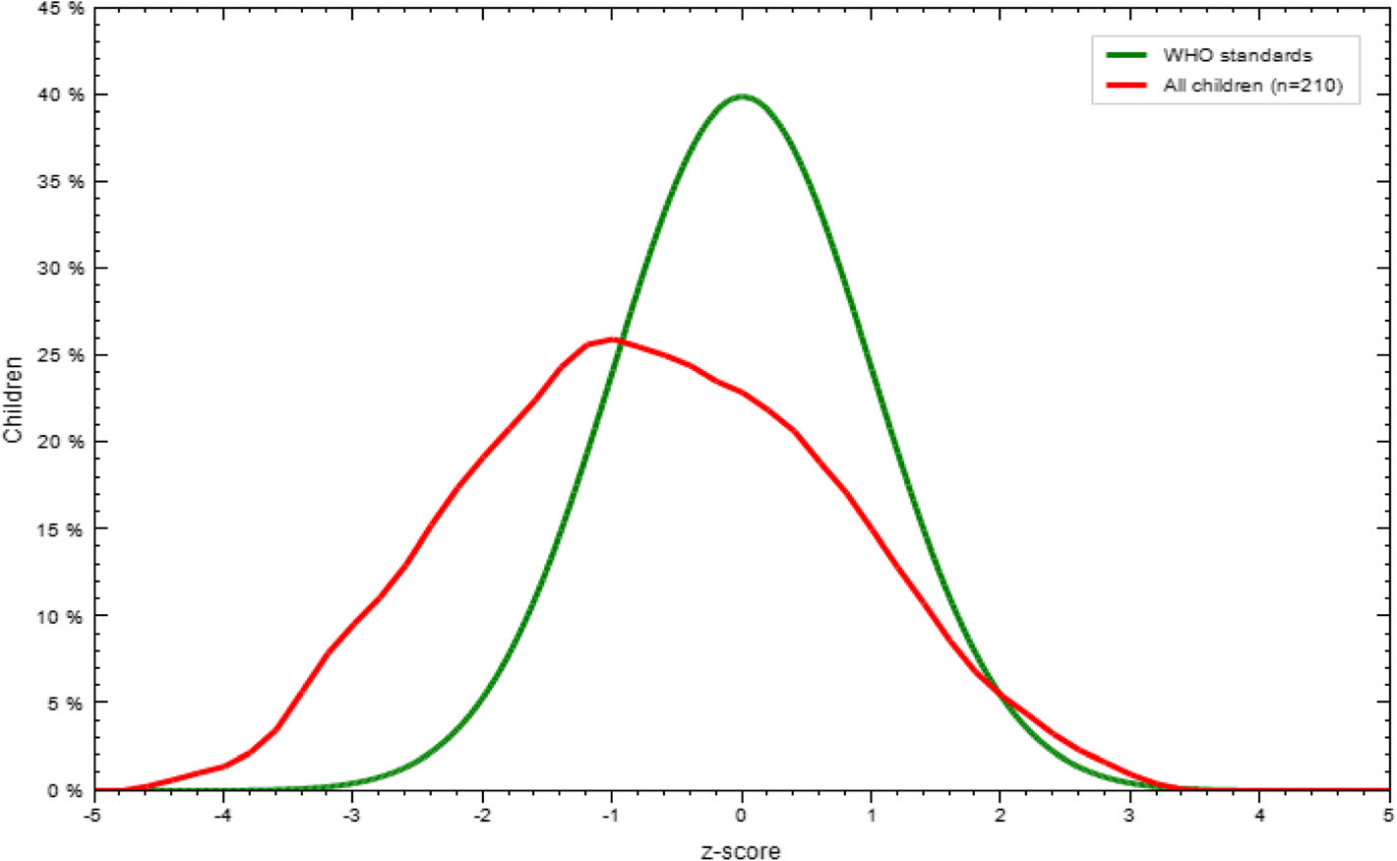
WLZ scores compared to WHO growth standards in Filtu town, Somali region, April 2013

**Fig. 2:**
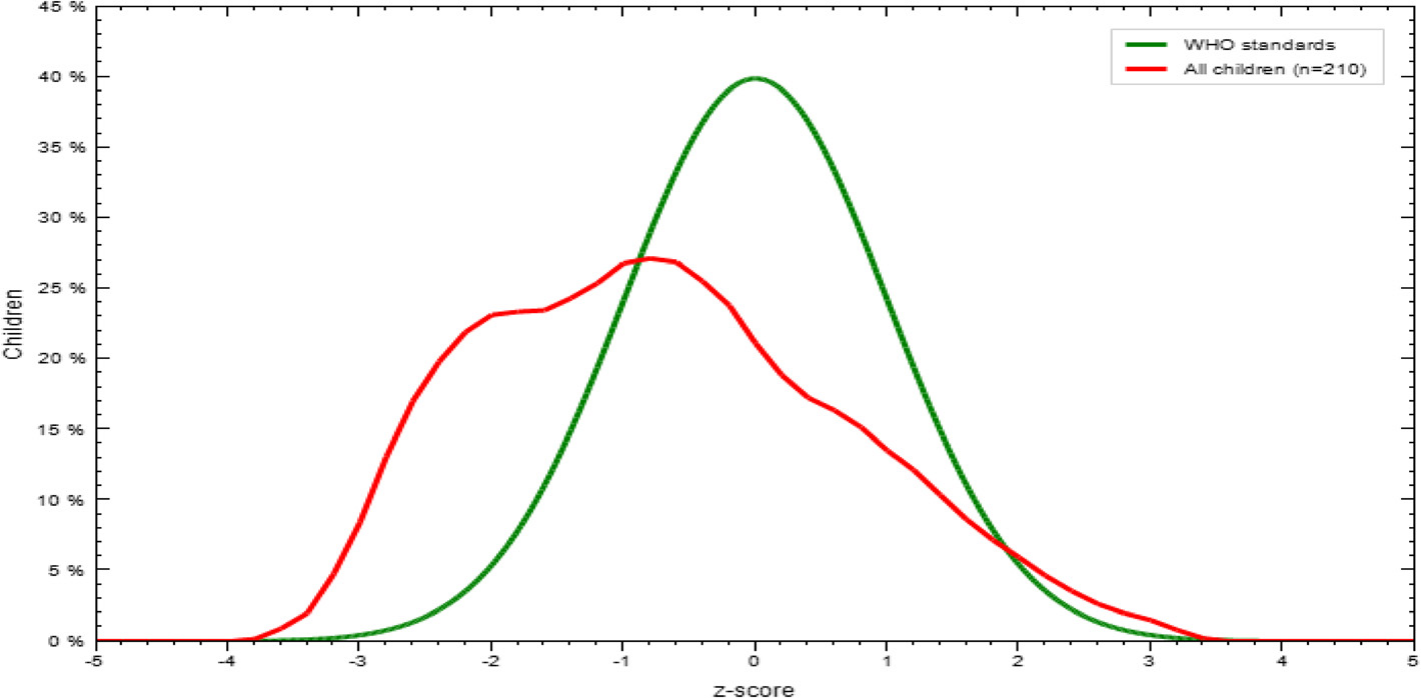
LAZ scores compared to WHO growth standards in Filtu town, Somali region, April 2013

**Fig. 3:**
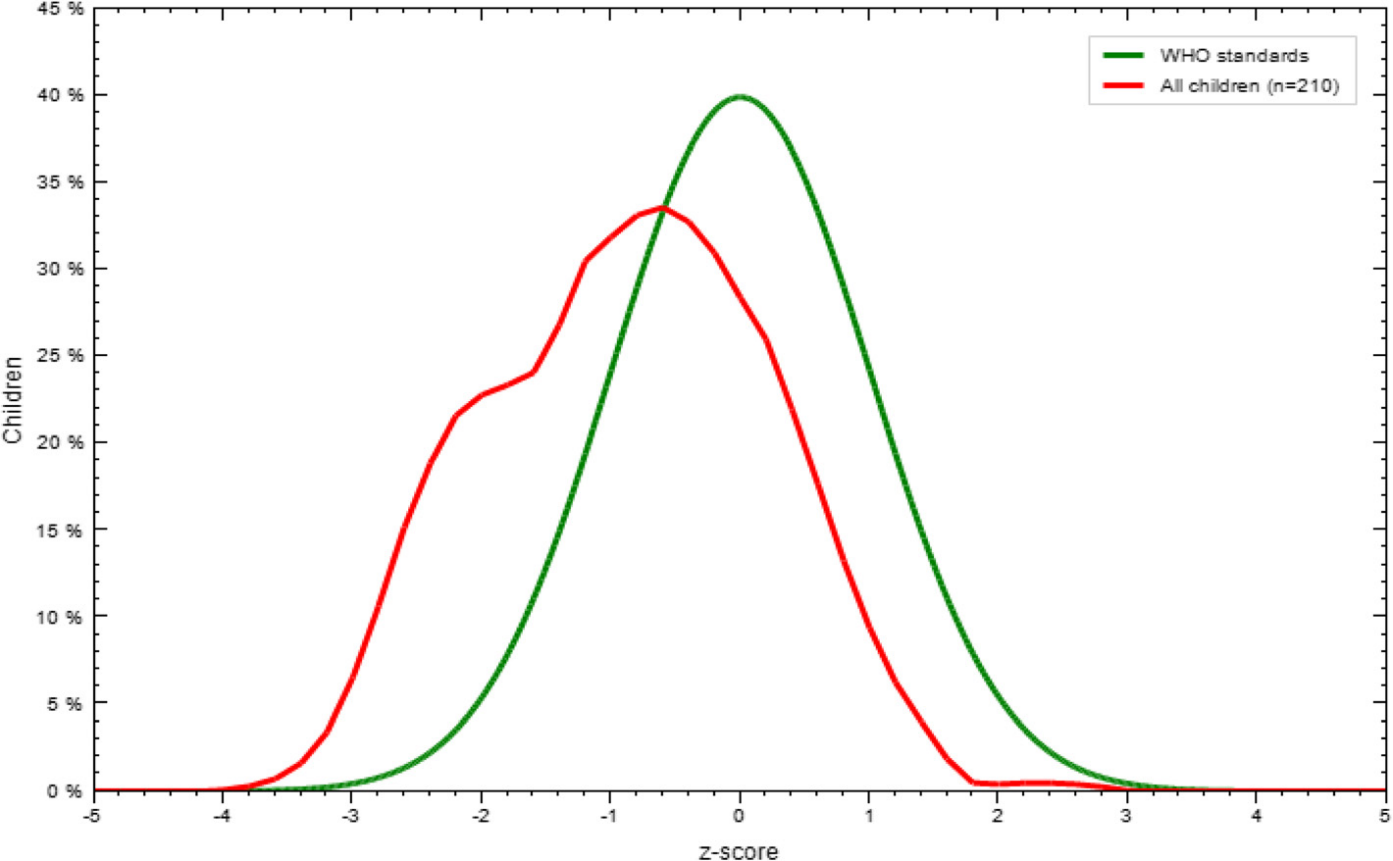
WAZ scores compared to WHO growth standards in Filtu town, Somali region, April 2013

The mean (±SD) of WLZ, LAZ and WAZ of the infants and young children of the study participants were −0.72 (±1.3), −0.68 (±1.33), and −0.86 (±1.06), respectively. As shown in Figs. 1, 2 and 3, Z-score curves are displaced to the left of the WHO growth reference curve demonstrating that malnutrition is prevalent among infants and young children in Filtu town. The prevalence of wasting, stunting and underweight among infants and young children in Filtu town was 17.5 % (95 % CI: 12.91-23.22), 22.9 % (95 % CI: 17.6-28.9) and 19.5 % (95 % CI: 14.58-25.3) respectively.

### Determinants of Nutritional Status

Among the variables entered into univariable (bivariate) logistic regression analysis, breastfeeding (COR = 0.32 (95 % CI: 0.13-0.77)) was associated with significantly lower odds of wasting, while bottle feeding (COR = 2.74 (95 % CI: 1.18-6.38)) and diarrheal disease within the past two weeks (COR = 2.52 (95 % CI: 1.23-5.19)) were associated with increased odds of wasting. The multivariable logistic regression model showed that recommended breastfeeding practices (AOR = 0.38 (95 % CI: 0.14-0.99)) and diarrheal diseases (AOR = 2.13 (95 % CI : 1.55-4.69)) were independent predictors respectively of reduced and increased odds of wasting (Table 2).

**Table 2:** Factors associated with wasting among infants and young children in Filtu town, Somali Region, April 2013

Characteristics		Number of wasted children	^a^ COR (95 % CI)	^b^ AOR (95 % CI)
Maternal education status	Never attended formal education	29	1.13 (0.85-1.48)	
	Attended formal education	8	1	
Food security status	Food insecure	6	1.46 (0.16-1.27)	
	Food secure	31	1	
Age at complementary food introduction			1.60 (0.76-3.38)	
Acute resp. infection in the last 15 days	Yes	9	1.62 (0.73-3.63)	
	No	28	1	
Diarrheal disease in the 15 days	Yes	20	2.52 (1.23-5.19)	2.13 (1.55-4.69)*
	No	17	1	
Recommended meal frequency	Yes	13	0.96 (0.82-1.11)	
	No	24	1	
Bottle feeding yesterday	Yes	28	2.74 (1.18-6.38)	2.32 (0.98-4.22)
	No	8	1	
Breast feeding in the last 24 hour	Yes	30	0.32 (0.13-0.77)	0.38(0.14-0.99)*
	No	7	1	
24 hour dietary diversity score	≥4	7	1.52 (0.62-3.69)	
	<4	30	1	
Wealth index	Poor	4	1.17 (0.25-5.47)	
	Medium	10	1.22 (0.35-4.74)	
	High	4	1	

* statistically significant at p value <0.05

^a^ Crude Odds Ratio

^b^ Adjusted Odd Ratio

In the univariable logistic regression model, bottle feeding, breastfeeding, 24 h diet diversity and age at complementary feeding initiation were significantly associated with stunting. However in the final multivariable model, the independent predictors of reduced odds for stunting were higher dietary diversity score (AOR = 0.45(95 % CI: 0.21-0.95)) and appropriate age (6 months) of complementary feeding initiation (AOR = 0.25(95 % CI: 0.09-0.66)). Bottle feeding was significantly associated with increased odds of stunting (AOR = 3.83(95 % CI: 1.69-8.67)) (Table 3).

**Table 3:** Factors associated with stunting among infants and young children in Filtu town, Somali Region, April 2013

Characteristics		Number of stunted children	^a^ COR (95 % CI)	^b^ AOR (95 % CI)
Maternal education status	Never attended formal education	38	1.24 (0.95-1.62)	
	Attended formal education	10	1	
Food security status	Food secure	2	6.25 (0.81-47.9)	
	Food insecure	46	1	
Age at complementary feeding introduction			0.27 (0.11-0.68)	0.25 (0.09-0.66)*
Acute resp. infection in the last 15 days	Yes	14	1.24 (0.62-2.46)	
	No	34	1	
Diarrheal disease in the 15 days	Yes	13	1.67 (0.82-3.39)	
	No	35	1	
Recommended meal frequency score	Yes	23	0.92 (0.76-1.06)	
	No	25	1	
Bottle feeding yesterday	Yes	34	5.53 (2.72-11.25)	3.83 (1.69-8.67)*
	No	14	1	
Breastfeeding in the last 24 hour	Yes	20	2.96 (1.53-5.74)	1.73 (0.78-3.82)
	No	28	1	
24 hour dietary diversity	≥4	22	0.27 (0.13-0.54)	0.45 (0.21-0.95)*
	<4	26	1	
Wealth index	Poor	4	1.56 (0.36-6.76)	
	Medium	15	1.07 (0.38-3.13)	
	High	5	1	

* statistically significant at p value <0.05

^a^ Crude Odds Ratio

^b^ Adjusted Odd Ratio

As shown in Table 4 the factor which had significant association with reduced odds for underweight was breastfeeding (AOR = 0.24(95 % CI: 0.1-0.59)). The factor associated with increased odds for underweight was diarrheal disease in the past 15 days (AOR = 3.54(95 % CI: 1.17-7.72)) (Table 4).

**Table 4:** Factors associated with underweight among infants and young children in Filtu town, Somali Region, April 2013

Characteristics		Number of underweight children	^a^ COR (95 % CI)	^b^ AOR (95 % CI)
Maternal education status	Never attend formal education	31	1.08 (0.83-1.40)	
	Attend formal education	10	1	
Food security status	Food secure	5	1.70 (0.24-2.05)	
	Food insecure	36		
Age at complementary feeding introduction			1.15 (0.55-2.41)	
Acute resp. infection in the last 15 days	Yes	12	1.22 (0.58-2.53)	
	No	29	1	
Diarrheal disease in the 15 days	Yes	22	3.96 (0.19-0.79)	3.54 (1.17-7.72)*
	No	19	1	
Recommended meal frequency score	Yes	16	0.93 (0.86-1.12)	
	No	25	1	
Bottle fed yesterday	Yes	26		
	No	12	0.64 (0.30-1.34)	
Breast feeding in the last 24 hour	Yes	34	0.27 (0.11-0.64)	0.24 (0.10-0.59)*
	No	7	1	
24 hour dietary diversity	≥4	10	0.99 (0.44-2.20)	
	<4	31	1	
Wealth index	Poor	4	1.17 (0.26-5.43)	
	Medium	10	1.22 (0.34-4.41)	
	High	4	1	

*statistically significant at p value <0.05

^a^ Crude Odds Ratio

^b^ Adjusted Odd Ratio

## Discussion

Appropriate breast-feeding and complementary feeding practices are fundamental to children’s survival, growth and development [12]. Continued, frequent breastfeeding also protects child health and wellbeing by reducing the child’s risk of morbidity and mortality in disadvantaged populations [9].

In this study 17.5 % (95 % CI: 12.91-23.22), 22.9 % (95 % CI: 17.6-28.9) and 19.5 % (95 % CI: 14.58-25.3) of infants and young children were wasted, stunted and underweight respectively. According to the 2011 EDHS, wasting prevalence for Somali Region for the specific age groups of 6–23 months children was 17.8 % [13] which was similar to our results in Filtu town. A study from North Showa, Ethiopia, reported that the prevalence of wasting, stunting, and underweight were 10.6, 54.2, and 40.2 % respectively [19]. In the current study, the prevalence of acute malnutrition (wasting) was found to be higher while the prevalence of stunting and underweight were relatively lower as compared to the study from North Showa, Ethiopia.

This might be due to differences in agro-ecology, feeding habits, life styles and demography between the two study areas.

As reported in the 2011 EDHS, the prevalence of wasting is higher among the pastoralist communities [13].An additional explanation could be that the data for this study were collected during drought season, when milk production, the most regarded complementary food, was scarce [15].

In the present study the associated factors of wasting and underweight were diarrheal disease and breastfeeding. Diarrheal disease was associated with higher odds of wasting and underweight while breastfeeding was found to be protective for wasting and underweight. Childhood morbidity status, especially diarrhea, has been reported in other studies to have a negative effect on growth of children, specifically on weight gain [20]. Diarrhea and other conditions such as fever affect both dietary intake and utilization, consequently affecting a child’s nutritional status [21]. Infection and malnutrition have always been intricately linked. Malnutrition is the primary cause of immunodeficiency worldwide [22]. The association between malnutrition and diarrheal diseases, as for most infections, is bidirectional; that is, the nutritional state alters the host response to infection and infectious illness alters nutritional state. Approximately 30 % of diarrheal deaths can be attributed to suboptimal breastfeeding [23]. Due to the cross-sectional nature of this study, it is difficult to know if the diarrhea results in poor nutritional status or if the diarrhea comes because of the poor nutritional status.

In this study breastfeeding was significantly associated with lower odds of wasting. However the precision of the AOR as indicated by the 95 % CI was low. This could be attributed to the small sample size and the small number of wasted children who were breastfed. There is evidence that breastfeeding was associated with weight gain [20, 24]. Breastfeeding is also associated with lower incidence of child morbidity [25–28]. This could be due to the immunological, hygienic and nutritional advantages of breastfeeding. These additional advantages of breastfeeding have a role in prevention of malnutrition.

Stunting was associated significantly with the variables including age at which complementary feeding commenced, bottle feeding, and dietary diversity score. Bottle feeding was identified as a factor significantly associated with higher odds of stunting. Complementary feeding initiation at recommended age (6 months) and better dietary diversity scores (≥4) were also associated with reduced odds of stunting. A study from Uganda showed that low dietary diversity was a predictor of stunting [29]. A study done in Sidama zone reported that dietary inadequacy and low diet quality in terms of diversified diet and availability of micronutrients had a significant negative association with child growth [30]. A report by Dishaand colleagues identified a strong association between low dietary diversity score and low LAZ score of infants and young children [24].

Maternal education was not statistically associated with any of the anthropometric indices. A study done in Zambia showed no significant association between maternal education status and stunting and underweight whereas wasting prevalence did have a significant association with maternal education in which wasting prevalence were minimal in households with educated mothers [31]. On the other hand, a study done in Nigeria and Uganda showed that there was a positive association between levels of maternal education and nutritional indices [29, 32]. A study from Jimma in Ethiopia reported no statistically significant association between child nutritional status and education status [33]. In our study 71.4 % of mothers had no education. This means the largest proportion of mothers would have similar child feeding behaviors because of lack of education. Mothers’ education affects proper child feeding practices through enhancing their knowledge from different information sources like newspapers by independent learning. But in this study area most of the mothers do not have access to any reading materials regarding child feeding. So having better educational status might not affect child nutritional status.

Factors like acute respiratory infection in the last 15 days, meal frequency, food security status and wealth index were not significantly associated with any of the nutritional indices. A study from Jimma also reported that there was no significant association between any of the nutritional indices and mothers’ age, education, religion, residence or the sex of the child [33].

This study has two limitations. The study employed a cross-sectional study design which could not establish a cause and effect relationship between the dependent and independent variables. The other limitation of the study is that it was done in an urban areas which may not represent pastoralists living in the rural areas.

## Conclusion

The prevalence of under nutrition is a public health problem, among infants and young children in Filtu town, Somali region, Ethiopia. Not breastfeeding during the previous day and diarrheal disease were found to be independent predictors of increased odds for wasting and underweight. Low dietary diversity score, inappropriate age of complementary feeding initiation and bottle feeding were identified to be significant predictors of stunting. Interventions to improve dietary diversity, breastfeeding practices, and timely initiation of complementary feeding are important to reduce under nutrition in Filitutown, Somali region. Besides those nutrition interventions, prevention of diarrheal disease and reducing bottle feeding would have important effects in reduction of under nutrition.

## Abbreviations

DHS: Demographic and Health Survey
FANTA: Food and Nutrition Technical Assistance Project
IYC: Infants and young children
IYCF: Infant and young child feeding
LAZ: Length-for-age Z score
UNICEF: United Nations Children’s Fund
WAZ: Weight-for-age Z score
WHO: World Health Organization
WLZ: Weight-for-length Z score

## References

[1] UNICEF and WHO. Global Strategy: Breastfeeding critical for child survival. Available at: http://www.who.int/mediacentre/news/releases/2004/pr19/en/index.html (date Accessd May 25, 2011) 2004. 15510403

[2] WHO. Infant and young child feeding: Available at http://www.who.int/mediacentre/factsheets/fs342/en/. 2015.

[3] WHO Multicentre Growth Reference Study Group (2006). Complementary feeding in the WHO Multicentre Growth Reference Study. Acta Paediatr Suppl.

[4] UNICEF Statistics. Progress for children: a child survival report card. Available at: http://www.cdc.gov/malaria/impact/index.htm. 2006.

[5] Guerrant RL, Schorling JB, McAuliffe JF, Souza MA (1992). Diarrhea as a cause and an effect of malnutrition: diarrhea prevents catch-up growth and malnutrition increases diarrhea frequency and duration. Am J Trop Med Hyg.

[6] Schorling JB, McAuliffe JF, Souza MA, Guerrant RL (1990). Malnutrition is associated with increased diarrhoea incidence and duration among children in an urban Brazilian slum. Int J Epidemiol.

[7] Yoon PW, Black RE, Moulton LH, Becker S (1997). The effect of malnutrition on the risk of diarrheal and respiratory mortality in children < 2 y of age in Cebu, Philippines. Am J Clin Nutr.

[8] Bryce J, Boschi-Pinto C, Shibuya K, Black RE (2005). WHO estimates of the causes of death in children. Lancet.

[9] WHO Collaborative Study Team on the Role of Breastfeeding on the Prevention of Infant Mortality (2000). Effect of breastfeeding on infant and child mortality due to infectious diseases in less developed countries: a pooled analysis. Lancet.

[10] Dewey KG, Brown KH (2003). Update on technical issues concerning complementary feeding of young children in developing countries and implications for intervention programs. Food Nutr Bull.

[11] Nancy K, Jamie W (2002). Zinc and breastfed infants: if and when is there a risk of deficiency?. Adv Exp Med Biol.

[12] Brown K, Dewey K, Allen L. World Health Organization: Complementary feeding of young children in developing countries: a review of current scientific knowledge: WHO reference number: WHO/NUT/98.1. Available at http://www.who.int/nutrition/publications/infantfeeding/WHO_NUT_98.1/en/. 1998.

[13] Central Statistical Agency (CSA) Ethiopia (2012). Ethiopia demographic and health survey 2011.

[14] Central Statistical Agency (CSA) [Ethiopia]. The 2007 population and Housing Census of Ethiopia. Statistical Summary Report at National Level, Addis Ababa, Ethiopia: Available at http://ecastats.uneca.org/aicmd/Portals/0/Cen2007_firstdraft.pdf. 2008.

[15] Sadler K, Catley A. Milk Matters: the role and value of milk in the diets of Somali pastoralist children in Liben and Shinile, Ethiopia. Feinstein International Center, Tufts University and Save the Children, Addis Ababa.: Available at http://fic.tufts.edu/assets/Milk-Matters-in-2009.pdf. 2009:30.

[16] Bruce C. Anthropometric Indicators measurement Guide. Food and Nutrition Technical Assistance Project, Academy for Educational Development, Washington DC: Available at http://www.ergo-eg.com/uploads/books/anthro_1.pdf. 2003.

[17] Ballard T, Coates J, Swindale A, Deitchler M. Food and Nutrition Technical Assistance III Project (FANTA): Household Hunger Scale: Indicator Definition and Measurement Guide: Available at http://www.fantaproject.org/sites/default/files/resources/HHS-Indicator-Guide-Aug2011.pdf. 2011.

[18] WHO. Indicators for assessing infant and young child feeding practices: Available at http://apps.who.int/iris/bitstream/10665/44368/1/9789241599757_eng.pdf. 2010.

[19] Aweke KA, Habtamu F, Akalu G (2012). Nutritional Status of Children in Food Insecure Households in Two Districts of North Showa Zone, Ethiopia. Afr J Food Agric Nutr Dev.

[20] Saha KK, Persson L, Rasmussen KM, Arifeen SE, Frongillo EA, Alam DS (2008). Appropriate infant feeding practices result in better growth of infants and young children in rural Bangladesh. Am J Clin Nutr.

[21] Mbori-Ngacha D, Otieno J, Njeru E, Onyango F (1995). Prevalence of persistent diarrhoea in children aged 3–36 months at Kenyatta National Hospital, Nairobi, Kenya. East Afr Med J.

[22] Katona P, Katona-Apte J (2008). The Interaction between Nutrition and Infection. Clin Infect Dis.

[23] WHO. Global health risks: mortality and burden of disease attributable to selected major risks: Available at http://www.who.int/healthinfo/global_burden_disease/GlobalHealthRisks_report_full.pdf. 2009.

[24] Disha AD, Rawat R, Subandoro A, Menon P (2012). Infant and young child feeding (IYCF) practices in Ethiopia and Zambia and their association with child nutrition: Analysis of demographic and health survey data. Afr J Food Agric Nutr Dev.

[25] Diallo F, Bell L, Moutquin J, Garant M (2009). The effects of exclusive versus non-exclusive breastfeeding on specific infant morbidities in Conakry. Pan Afr Med J.

[26] Story L, Parish T. Breastfeeding helps to prevent two major infant illnesses The internet Journal of the Allied Health sciences and practices. 2008;6(3):2–5.

[27] Lamberti LM, Walker CLF, Noiman A, Victora C, Black RE (2011). Breastfeeding and the risk for diarrhea morbidity and mortality. BMC Public Health.

[28] Koyanagi A, Humphrey JH, Moulton L, Ntozini R, Mutasa K, Iliff P, Black R (2009). Effect of early exclusive breastfeeding on morbidity among infants born to HIV-negative mothers in Zimbabwe. Am J Clin Nutr.

[29] Bukusuba J, Kikafunda JK, Whitehead RG (2009). Nutritional status of children (6–59 months) among HIV-positive mothers/caregivers living in an urban setting of Uganda. Afr J Food Agric Nutr Dev.

[30] Gibson RS, Abebe Y, Hambidge KM, Arbide I, Teshome A, Stoecker BJ (2009). Inadequate feeding practices and impaired growth among children from subsistence farming households in Sidama, Southern Ethiopia. Matern Child Nutr.

[31] Masiye F, Chama C, Chita B, Jonsson D (2010). Determinants of child nutritional status in Zambia: An analysis of a national survey. Zambia Soc Sci J.

[32] Lawal BO, Samuel FO (2010). Determinants of nutritional status of children in farming households in Oyo State, Nigeria. Afr J Food Agric Nutr Dev.

[33] Beyene TT (2012). Predictors of nutritional status of children visiting health facilities in Jimma Zone, South West Ethiopia. Int J Adv Nurs Sci Pract.

